# The characteristics and role of digital literacy in an effective health protection

**DOI:** 10.1016/j.heliyon.2024.e29737

**Published:** 2024-04-15

**Authors:** Predrag Bejaković, Željko Mrnjavac

**Affiliations:** a Independent researcher, Zagreb, Croatia; b The Faculty of Economics, Business, and Tourism, University of Split, Croatia

**Keywords:** Healthcare service, Health literacy, Telemedicine, P5 technologies

## Abstract

The evolution of modern healthcare underscores the crucial question of societal priorities in development. When distributing finite healthcare resources, decision-makers must weigh numerous factors and navigate a range of sometimes conflicting criteria, including equity, access, fairness, and effectiveness. This chapter aims to survey the social implications of digital literacy as a pivotal element for the optimal advancement of P5 medicine, with the objective of enhancing healthcare services. The untapped potential of telemedicine and other emerging P5 technologies remains substantial in terms of their integration into routine medical practice. Adequate digital literacy among both healthcare providers and users stands as a vital prerequisite for the successful adoption and further evolution of these innovations. Coupled with improved health literacy, these factors collectively should contribute to the provision of superior healthcare services and consequently to the overall well-being of the populace.

## 1 Introduction

Health is essential to the well-being of a person, and it is also a prerequisite for a prosperous and fecund society. A healthy population and particularly a labour force are important for economic growth and sustainable economic and social development. However, there is no guarantee that economic development and growth necessarily improve general health and well-being. Instead, health related problems and illness can endanger production and consumption, thwart recreation and travel, and therefore cause a deterioration of general welfare.

Enhanced health conditions bolster economic growth through four potential avenues: reducing productivity losses stemming from employee illness and absenteeism, unlocking previously untapped natural resources due to disease constraints, boosting school enrolment and facilitating improved learning for children, and freeing up financial and human resources that would otherwise be allocated towards treating illnesses for other purposes [1]. Serious and long-term diseases often affect the productivity of people and their possibility to accumulate human capital, required for successful employment and work [2]. The economic benefits of improved health conditions are relatively greater for the population in developing countries and for the poor people in rich countries, who usually more suffered from poor health condition.

Existing healthcare systems organising patient healthcare in the traditional way - where patients are treated either in outpatient medical practices or in hospitals - are more and more limited regarding huge costs and/or insufficient human resources. Alternatives such as managed care models and broader implementation of P5 medicine - predictive, preventive, personalised, participatory and psycho-cognitive medicine - which guide patients through the healthcare system, are already having an increasing role. This role should get on importance in the nearly future. Recently, digitalization as well as new medicine technologies have entered the field with great hope. They are seen as potential tools for optimizing existing processes in the healthcare system, with the expectation of implementing new ways of providing healthcare. In telemedicine, for example, medical services in the field of consulting, diagnostics, and therapy are provided on the very long distances or time differences using diverse information and communication technologies.

The full potential of telemedicine, along with emerging P5 technologies, in terms of their utilization and integration into conventional medical practice, remains untapped. Medicine will become proactive in nature and it will be more and more oriented towards improving health conditions, rather than curing disease. Within a decade, patients will be encircled by a virtual cloud of enormous quantity of data and with the help of artificial intelligence doctors will have the possibility to reduce them into simple hypotheses about how to optimise wellbeing and how to prevent diseases. This is really a revolution in healthcare and “just as the development of moveable type transformed the world by making the Bible available to the masses, the development of modern telecomputing is creating a Reformation in many domains - including healthcare” [3]. Such healthcare has the opportunities to revolutionary change needed medicine provision in a way that offers a range of challenges to the existing structures and processes of traditional healthcare delivery.

In order to maximize the advantages of information and communication technology (ICT) and realize the developmental potential of the digital age, nations must address the prevalent digital divide, which stands as one of the most pressing and significant challenges confronting the information society.

This article through narrative literature review explains the role of digitalization and digital literacy on successful application of P5 technologies and telemedicine as well as on improving health literacy in achieving successful healthcare provision. After this introduction remarks, follows Section 1 dedicated to the importance and the role of the digital literacy in provision of healthcare services. Section 2 deals with the characteristics and possibilities of P5 technologies and telemedicine. Section 3 explains a link between digitalization and health literacy. Conclusions and recommendations are presented in Section 4.

## 2 The role of the digital literacy in provision of healthcare services

Emerging technologies are revolutionizing healthcare delivery in various capacities and for diverse objectives. For instance, internet-based appointment scheduling has become widespread, allowing patients to view their physician's schedule and select suitable appointment times. The storage of patient data online empowers healthcare service users to take ownership of their electronic medical records. Online platforms and websites dedicated to health data enable patients to document their medical history and track their health status over time. With the exponential rise in internet usage, social media engagement, and the adoption of mobile health applications and wearable sensors, the impact of digital health in supporting patient engagement and activities, as well as enhancing access to healthcare, is rapidly accelerating. Advancements in computational analysis of data present an unprecedented opportunity to explore and comprehend disease-specific genetic variations and relevant social determinants, thereby enabling personalised treatment and prevention strategies [4].

The World Health Organization [5] defines a digital health as “the combination of e-health and m-health as well as emerging areas, such as the use of advanced computing sciences in big data, genomics and artificial intelligence.” For successful use of all available digital and informational possibilities, there is a need that health providers and patients have at least a basic digital literacy. Digital literacy encompasses the skills necessary for the safe and discerning utilization of electronic media in professional settings, leisure activities, and communication. These competencies are linked to logical and critical thinking, proficient information management, and effective communication abilities. They entail leveraging multimedia technology to locate, acquire, store, create, present, and distribute information, as well as to engage in online communication and participation. t, but still, 46% of Europeans, particularly older individuals, currently lack basic digital skills. This deficit impedes their ability to utilize digital technologies for everyday tasks and access online services [6]. As digital technologies transform textual information more and more into visual media so traditional skills like visual literacy are changing and becoming crucial part of digital literacy [7], but all other related literacies are changing as well The realm of digital literacy becomes increasingly challenging to definitively define with the continuous emergence of new digital technologies, tools, and literacies. The concept of digital literacy has roots dating back to the 1960s, and it has evolved over time. This evolution is detailed in the comprehensive discussion of the changing definitions of digital literacy overtime by Reddy et al. [8].

Due to continuous developments digital literacy is in more recent times conceived as “a combination of technical-procedural (e.g., handling files and editing visuals), cognitive (e.g., the ability to intuitively decipher or “read” visual messages embedded in graphic user interfaces, working with search engines, evaluating data, sorting out false and biased data, and distinguishing between relevant and irrelevant data) and emotional-social (e.g., ability to communicate online safely, being aware of the legal and ethical guidelines of using digital platforms) skills.” [9].

According to New Media Consortium [10] digital literacy is “the set of abilities and skills where aural, visual, and digital literacy overlap. These include the ability to understand the power of images and sounds, to recognize and use that power, to manipulate and transform digital media, to distribute them pervasively, and to easily adapt them to new forms”.

Digitisation meaningfully changes present jobs, transforming to numerous degrees job contents and requirements. As it influences the way the working tasks are realised it requires the changes in the skills, knowledge and experiences [11]. People have a reasonable advantage over machines regarding cognitive tasks which entail flexibility to adapt and implement innovative “solutions, thinking and resolving unexpected problems” [12]. Furthermore, in many economic sectors and service provisions, particularly in such sensitive activities as healthcare, beneficiaries will always prefer personal contacts with humans than that with the machine. While digital health holds promise in addressing challenges like distance and access to healthcare, it also grapples with many of the systemic issues encountered by healthcare systems overall. These include issues such as suboptimal governance, inadequate training, limitations in infrastructure, and inadequate access to necessary equipment and supplies. These deliberations “need to be addressed in addition to specific requirements introduced by digital health. As the context will moderate the eventual impact of digital health interventions, the broader health system and enabling environment become especially critical” [5].

Digital literacy and skills are needed in more and more occupations in the provision of healthcare services. They have become crucial transversal competences. These skills are a group of attitudes, values and procedures, that can be without problems convey from one professional field to another. With the relentless progress of digital technology and the healthcare services based on it, digital literacy and skills have to be constantly updated, to circumvent or minimise the risks of digital exclusion. Erstad [13] deems that participation in the digital area and a society is no longer an issue of “have” or “have not”, but rather a question of the needed digital knowledge and skills.

Refining the notion of digital competencies, several scholars [[14], [15], [16]], delineate its components and constituent elements while specifying the skills it encompasses. Their aim is to facilitate the assessment of competencies and skills and enhance understanding of this phenomenon within society. Generally, digital literacy and skills encompass a variety of interconnected concepts. Curtarelli et al. [17] identify three primary categories of digital skills, which can be applied across various sets of abilities and are linked to the capability to undertake demanding and intricate tasks. The initial category comprises fundamental digital literacy competencies and skills necessary for digital literacy. The second category encompasses digital skills pertinent to employment, whereas the third category pertains to digital skills essential for ICT professions, required for the creation and advancement of new digital solutions, products, and services.

With the aim of improving the current situation, the European Commission has established the Digital Skills and Jobs Coalition [18]. This initiative assists Member States (MS) in cultivating a digital talent pool and ensuring that both the general populace and the workforce in Europe possess appropriate and up-to-date digital skills and competences. Despite the prioritization of digital skills across policy-making actions by this Coalition, comprehensive action remains limited in a significant number of MS. Furthermore, many countries lack a comprehensive strategy and/or fail to sustain continuous attention to the issue across various policy domains. Typically, efforts are made to align the education system with the demands of a knowledge-based economy, yet in many MS, insufficient emphasis has been placed on ICT practitioner skills and competences [19], and particularly to health worker competence in the provision of healthcare services [5].

Campanozzi et al. [20] seek to assess, through a review of the literature from the past decade, the influence of digital literacy on access to telemedicine services. Their objective is to determine whether high or low levels of digital literacy play a role in determining the utilization of telemedicine services and to what extent. The authors suggest that it is essential not only to implement digital education programs aimed at bridging the digital divide but also to plan for evaluation studies to assess the effectiveness of such programs in the near future.

Nazeha et al.'s review [21] aims to identify and assess existing digital health competency frameworks for healthcare professionals, offering recommendations for future digital health training initiatives and framework development. The authors conducted a comprehensive literature search to compile digital health competency frameworks published since 2000. They searched a total of 6 databases, including OpenGrey, ResearchGate, Google Scholar, Google, and relevant association websites. The review encompassed 30 frameworks, primarily targeting nurses and originating from high-income countries. Thematic analysis revealed 28 digital health competency domains across the included frameworks, with the most prevalent domains focusing on basic information technology literacy, health information management, digital communication, ethical and legal considerations, as well as data privacy and security. The majority of competencies are applicable to a broad spectrum of healthcare professionals. The authors suggest that digital health training initiatives should tailor competencies to specific healthcare worker groups, roles, seniority levels, and practice settings. The identified competency domains represent fundamental interprofessional competencies crucial for inclusion in healthcare workers' training and education.

Keane [22] states the importance of providing better education to patients and the wider audience. There is also a need to improve and modernise healthcare professionals’ education in holistic thinking and communication. The author announces the realisation of the research on digital skills and competences of persons employed in healthcare sector in few EU MS as well as in Serbia, whose results had to be available at the end of 2021 or at the beginning of 2022.

## 3 The digital literacy and possibilities of P5 technologies and telemedicine

There are various reasons why P5 medicine is expected to be predictive, preventive, personalised, participatory and psycho-cognitive. One of the most important reasons is an improved exploration and understanding of DNA, genes and the complete human genome. P5 is the new integrative concept in the healthcare service provision that represents a shift away from the model of reacting to illness to the model of prevention and maintaining health. It allows prediction of individual predisposition before the beginning of a disease in order to provide targeted preventive measures and to produce personalised treatment algorithms attuned to the person's health situation and needs. As elements of P5 medicine, machine learning, artificial intelligence, signal and image processing and telemedicine devices and communication are being widely used. Telemedicine enable a closer relationship with the patient by, for example, recording the patient's heart rate and by sending recommendations and alarms to patients in particular cases. All these tools in medicine, focused on the data related to patients, enable a patient-centered approach [23].

*Predictive medicine* means the use of laboratory and genetic tests to predict the initiation of a disease and/or worsening or amelioration of current disease [24]. The main goal of predictive medicine is to calculate a person's individual risk for any disease and intervene appropriately. This is accomplished by gathering and analyzing existing data to anticipate an individual patient's risk for a specific outcome, forecasting which treatment will be most efficacious for that individual, and intervening preemptively before the outcome manifests [25]. Earlier it was supposed that genetics would revolutionize medicine. Genetics and genomics have really significantly improved our ability to predict individual risk for some diseases and forecast which treatment will be more useful for a specific person. Nevertheless, genetics-based risk prediction has been of limited benefit because many current diseases are multifactorial, caused not only by genetics, but also by factors like age, diet, exercise and stress levels, as well as by environment or lifestyle, whose influence is not possible to assess by genetic testing. Furthermore, the validity and reliability of genetic testing are not 100%. To improve genetic testing, better biomarkers or biological markers are constantly developed and used. A biomarker as a measurement shows an interaction between a biological system and a potential hazard, which may be caused by various biological, chemical or physical factors. The further development of predictive medicine in the near future will be based on a wider use of genetic testing for more people and/or more disorders. Effective adoption and implementation will entail substantial efforts to not only purchase, develop and refine the requited IT infrastructure, but also to enable through training and education the health workers to achieve needed level of digital literacy for successfully use of the next generation of data-based medicine.

*Preventive medicine* aims to identify and address deviations in the health of individuals long before symptoms of disease manifest, thereby optimizing individual well-being and mitigating the onset of illness. It encompasses a range of strategies for disease prevention and health promotion, rather than solely focusing on symptom management and treatment. Just as health comprises various physical and mental states, disease and disability are influenced by lifestyle, environmental factors and genetic predisposition. Over the past approximately three decades, significant advancements have been made in preventive medicine. Some of these achievements stem from ongoing progress in scientific research, while others have been prompted by pressing public health issues and changes in healthcare services, both preventive and curative. Many improvements have been incremental, reflecting an evolutionary process, while others represent fundamental leaps forward in human understanding and its application in preventive medicine. Thus, Wallace [26] underlines the increased integration of business and administrative practices - primarily quality improvement techniques, measures or results and complex accounting - into prevention and public health service delivery. Furthermore, the increased role of public health education and programme delivery is important, as well as a wider acceptance of community health measures. The role of practicing professionals and their knowledge in preventive medicine is, however, not always clearly recognised. Nonetheless, various specialists, particularly epidemiologists, with adequate digital knowledge and skills, should be included in the execution of every essential task of the profession [23]. Evolving new infections, homicides, suicides, traffic injuries, workplace fatalities, adverse and persisting problems in cardiovascular diseases and many other ailments continue to limit the quality of life. The survey should uncover previously unknown risk factors and ineffective prevention measures. Effective communication and a full resolution of the ethical questions will be increasingly important to preventive medicine as it directly influences the behaviour of persons, the legal framework and it opens many ethical problems [26].

*Personalised medicine* involves tailoring medical care to individual patient differences across all stages, from prevention and diagnosis to post-treatment recovery. It encompasses the customization of diagnoses, medical treatments, and healthcare products for each patient [27]. Personalised medicine is a systematic use of patient-specific information with the purpose of selecting and implementing optimal prevention and/or wellbeing therapy [28]. The common goal of personal medicine is to boost “the targeting of diagnostic or therapeutic interventions in order to improve the clinical and cost effectiveness of healthcare provision” [29]. The overarching goal of personalised medicine is to refine the targeting of diagnostic and therapeutic interventions, thereby improving the clinical and cost effectiveness of healthcare delivery. Key features of this approach include prioritizing prevention and advancing the development of more effective drugs and treatment procedures. The focus is on facilitating innovative treatments while minimizing adverse side effects [30]. The implementation of personalised medicine is envisioned to progress through three phases, as outlined by Pavelić et al. [28]. The initial phase involves education, regulatory framework development, stakeholder engagement, and standardization, alongside dialogue with users. The second phase emphasizes harmonization, the establishment of molecular and environmental interaction networks, data integration, and ongoing monitoring. The third phase entails systematic data collection and the implementation of nanomedicine. Throughout all phases, digital education will play a pivotal role.

*Participatory medicine* is an architype shift, where patients cease to be only inactive passengers and become responsible chauffeurs of their health and where healthcare providers inspire and value them as full partners. Such attitude is currently accepted only by a minority of patients and physicians, but for its broader acknowledgement there is a need for more active advocacy and implementation of tools which enable changes in the culture of healthcare [31]. To achieve a real participatory healthcare system, major organisational, technical and societal obstacles have to be conquered. This will require a close collaboration with systems medicine - the application of systems biology approaches to disease - and big data [32], for which are required adequate digital skills.

*Psycho-cognitive medicine* emphasizes the importance of treating patients as individuals rather than passive recipients of care. Patients are evaluated based on their emotions, attitudes, and cognitive processes, which are closely linked to and significantly influence the healing process. This approach challenges the traditional evidence-based medicine model, which primarily relies on clinical trial results to determine optimal medical procedures and interventions. In contrast, psycho-cognitive medicine advocates for a healthcare model that considers the psychological and cognitive profile of each patient, rather than solely relying on diagnostic classifications. Achieving desired outcomes requires the development and implementation of new psychometric instruments dedicated to providing a comprehensive medical profile of the patient. Additionally, active promotion and respect for the patient's decision-making regarding their healthcare process are crucial aspects of this approach [33].

*Telemedicine* - enables healthcare specialists to assess, diagnose and treat patients from a distance by using modern telecommunications technology. The method has significantly evolved in the last 20 years or so and it is becoming an important part of the healthcare infrastructure. The age of the internet enabled profound changes for the application and accessibility of telemedicine. The spread of smart devices with the possibilities of high-quality video transmission, enabled delivery of remote healthcare services to patients in their homes or workplaces. Telemedicine involves utilizing electronic communication equipment and software to deliver clinical health services without the need for an in-person visit. This technology is commonly employed for follow-up appointments, chronic condition management, specialist consultations, and a range of other healthcare services that can be administered remotely through secure video and audio connections. As an alternative to in-person visits, telemedicine has many benefits for patients and health service providers. Patients spend less time away from work, they do not have travel expenses, enjoy more privacy and there is no exposure to other possibly infectious patients. Healthcare service providers can improve their efficiency, have better patient follow through and improved health outcomes and suffer from fewer missed appointments and cancellations.

There is almost no need to repeat the importance of a basic digital as well as health literacy for successful implementation of telemedicine and P5 technologies. Without a doubt, although there is an obvious similarity between digital and health literacy, their relation is not fully exploited and in the following text there are some ideas for improving health literacy as a mean for better health outcome.

## 4 Digital and health literacy

The digital transformation of healthcare can be disruptive due to the implementation of technologies such as the Internet, P5 technologies, telemedicine, virtual care, remote monitoring, artificial intelligence, big data analytics, blockchain, smart wearables, and others. These technologies enable and necessitate data exchange and storage, facilitate remote data capture, and promote the exchange of information across the healthcare ecosystem. Together, these factors create a continuum of care with the potential to enhance healthcare outcomes by improving medical diagnosis, digital therapeutics, treatment decisions based on clinical trial data, self-management of care, and person-centered care. Additionally, they contribute to the generation of more evidence-based knowledge, skills, and competencies among professionals, thereby supporting the provision of healthcare services [34].

Healthcare professionals and the social environment are important motivators in the process of increasing digital and health literacy. To increase the opportunity, people should have online access to useful information anytime and anywhere. To increase the ability and motivation, important factors are information, skilling and education [22]. The model developed by Carrasqueiro [35], defines in details a total of 37 healthcare professional's profiles grouped into three main categories: a) non-health - administrative and management professions involved in the management and support of eHealth services; b) health - clinical professions involved in the delivery of eHealth services; and c) ICT profiles involved in the management, development, operation and support of eHealth services.

Even educated persons with high literacy skills may face problems in obtaining, comprehending and using health information. Moreover, despite an increased interest for health literacy, almost no research has been realised on health professionals’ knowledge of health literacy and/or understandings of the obstacles to health literacy that patients have during the healthcare treatment [36]. The mysterious language and difficult to comprehend phrases used by professionals in health sector are fully incompressible for many patients. Furthermore, the unnecessarily complicated language and unclear scientific terms cause the users of healthcare sector to inadequately or wrongly understand words by healthcare professionals and the industry.

Adults who have a problem comprehending written materials are often embarrassed and apply methods to hide their difficulties. They are often not willing to demand explanation because they are scared of being considered illiterate and ignorant. If health workers were able to ask their patients to explain clearly and precisely what they understood about the diagnoses, instructions and drug information, they would find many gaps in the knowledge, grave problems in understanding, and widespread misinterpretations. These problems are also exacerbated by language and cultural variation, technological complexity in healthcare and present administrative documents and compound requirements.

Williams et al. [37] deem that functional health literacy is the ability to understand quantitative information, which often differs from the ability to read a prose text. According to Ratzan and Parker [38], health literacy is the degree to which a person has the capacity to process and understand basic health information and services needed to make appropriate health decisions. However, Nielsen-Bohlman et al. [39] state that health literacy is not just the possibility to understand basic information, but it appears when the expectations, preferences and skills of person receiving health information and services meet the expectations, preferences and skills of those providing them. They define health literacy as the possibility of patients to understand and to act in their own interest. Shohet and Renaud suggest a need for a holistic approach to health literacy, while at the same time focusing beyond the individual to consider the roles of organisations, health contexts and healthcare systems.

Health literacy remains an important neglected issue in achieving high-quality healthcare, but there are signs of improvement. More and mode health and medical experts with educational specialists create the methods of an accessible and understandable transfer of demanding health information to the patients and general public. The resistance towards technological changes and improvements can be lessened by improving the health literacy of patients. Due to the importance of health literacy, it is crucial to include health promotion and patient education in the preparation and realisation of different healthcare programmes [40]. Improvement in the field of health literacy requires the inclusion new measures which can be useful in establishment of baseline levels and monitor change during a longer period [39]. Literacy can be improved by formal individual programmes, but also innovative population-based approaches may also be very useful. Moreover, useful are mass public media campaigns which include television, radio, newspaper and billboard advertisements.

With the intention to lower system blocks, Lambert et al. [36] examine health professionals' understanding of health literacy and perceptions of barriers that their patients face in receiving healthcare services. The goal of the educational programme for health professionals’ should be focused on support them in provision of easy and comprehensible information to patient, so that they can better understand illness prevention, diagnoses, cures and instructions. In many countries, regular mass media health literacy programmes have improved eating habits and increased physical activity, and contributed to better management of chronic and degenerative diseases. Particularly for older population, health literacy can be improved by improving their self-management skills, strengthening their links to clinical care and by insuring ongoing efficient and adequate social support [41]. If the health information given to patients reflects real-life situations and is prepared in a simple and easily comprehensible language, according to Jacobson et al. [42], there is a significant possibility to improve the uptake of preventive health interventions, like vaccination against various pandemics.

In the measures for improvement of the health literacy, one should not neglect the role of digital literacy. While language and literacy skills differ in various people, there is a need to make a more careful effort to capitalize on what people can do rather than complain about the skills and literacy they do not have. As a starting point, measures could include integrating health literacy strategies and communication tools into medical training curricula. Such actions might contribute to improving physicians’ awareness of the health literacy problematic among patients, as well as to practicing techniques to enable effective interpersonal communication with patients, particularly those of the identified risk groups.

We are all accustomed to the use of digital applications in various aspects of our daily lives, so it is not surprising that they have expanded into the realm of healthcare as mobile health (mHealth) apps. These apps have the potential to enhance access to care, offering a low-cost intervention that requires minimal staff training or physical presence, thereby increasing access for those who are geographically isolated. However, a challenge arises from the fact that many mHealth apps are commercial products lacking an evidence base. Additionally, concerns persist regarding the safety and privacy of data transmitted through these apps [43]. This shows that digital tools can be transformative in providing better health care, but require that health workers and patients are respectively trained to understand and be aware of all effects of digitalization.

## 5 Conclusions and recommendations

Digital literacy has become a prominent topic in policy discussions and research concerning healthcare services. Collaborative efforts among key stakeholders, including service providers, users, government agencies, interest groups, insurance institutions, educational entities, media outlets, and others, are expected to result in action plans informed by scientific inquiry. To identify current and future digital literacy needs across various groups and society as a whole, it is essential to gather data on skills development and analyze user trends.

Digital literacy encompasses the fostering of creativity and educational methods adaptable to change. The influence of social media on learning within formal and informal education settings may offer insights into the components of digital literacy. Advancing digital literacy contributes to the establishment of a knowledge-based society, empowering individuals to comprehend, process, and utilize vast amounts of information effectively. Acquiring digital literacy equips individuals with the tools and experience needed to cultivate adaptable technological skills crucial for future professional endeavors. Ensuring widespread digital literacy, skills, and connectivity in today's digital landscape is a significant challenge for all involved parties, particularly in the provision of accessible healthcare services essential for individual well-being and a competitive economy. To address current and future digital literacy needs in society and the economy, comprehensive data on skills development and user trends are imperative. Achieving these objectives necessitates robust and collaborative partnerships at both the European and national levels, where stakeholders work together to bridge the existing digital skills gap.

According to various sources (primarily OECD, 2015; 2016; [5,34,44,45]), in such process there is a need to take care of.

• Effectiveness - The evidence from digital education and training indicates that this intervention enhances health workers' knowledge. However, the impact of this intervention on other outcomes, such as health workers' performance, skills, and attitudes, remains uncertain due to either a lack of direct evidence or evidence of very low certainty.
• Content - For the development of effective digital and health literacy there is a need to provide adequate training for health professionals. Targeted education and training programmes are important tools to solve low literacy awareness and understanding among health professionals. Such programs should incorporate basic and advanced information and theory about digitalised and health literacy. Furthermore, it is important to ensure the development of user-friendly information adjusted to low digital and health literate patients and their particular health condition. Such content should be given in a mode which minimise system barriers, for example time restraints and unnecessarily medicalised language.
• Acceptability - The qualitative evidence from medical and nursing students show that users respect digital educational and training, particularly advantages to mobile learning tools. This include the ease and portability of accessing materials and ability to personalize and adjust content to once own needs.
• Accessibility - Health workers located in remote areas (for example, mountains or islands) and rural communities find the digital training and provision of support very helpful in overcoming geographical barriers and linking to the broader health system. However, some health workers in these settings may have lower levels of training and digital literacy, what can be a serious obstacle for their regular participation in education and use of available possibilities. There is a need to insure adequate accessibility of digital health education and support frameworks also to underdeveloped parts of the MS as well as to low- and middle-income countries.
• Accuracy and adequacy - The systematic review of mobile learning and mobile support for healthcare services highlights the importance of continuously updating digital health education and support frameworks with emerging digital health technologies. When developing educational, training, or skilling programs, it is essential to target specific competencies relevant to particular groups of healthcare workers, as well as essential interprofessional competencies that should be integrated into training efforts for all healthcare worker groups. Educators should consider incorporating digital health training and skilling into existing parts of the curriculum and delivering it in a practical, applied manner whenever possible. Supporting digital skills of the health workforce is even more important and needed in the circumstances of the constant changing nature of healthcare systems and healthcare delivery.

The advancement of P5 medicine will hinge significantly on the integration and utilization of diverse datasets to construct comprehensive personal healthcare records, along with continued enhancements in digital and health literacy. In the foreseeable future, P5 medicine is likely to offer the prospect of genome modification by identifying the root cause of particular illnesses and modifying the human genome accordingly. This approach holds the potential to reduce the likelihood of diseases stemming from adverse environmental factors and lifestyle choices. Desired results can be achieved by educational programmes which contribute to improving physicians’ awareness of the health literacy problematic among patients, particularly those of the identified risk groups. Upgraded health literacy and further development and implementation of P5 medicine could be a really useful mixture to improve healthcare outcomes.

## Data availability

There are no data associated with this study deposited into a publicly available repository, as all information and sources were included in the paper and referenced.

## Funding

Contribution of Predrag Bejaković is correctly linked to project funded by European Social Fund UP.04.2.1.06.0055.

## CRediT authorship contribution statement

**Predrag Bejaković:** Writing – review & editing, Writing – original draft, Validation, Investigation, Formal analysis, Data curation, Conceptualization. **Željko Mrnjavac:** Writing – review & editing, Writing – original draft, Validation, Investigation, Formal analysis, Data curation, Conceptualization.

## Declaration of competing interest

The authors declare the following financial interests/personal relationships which may be considered as potential competing interests: Predrag Bejakovic reports financial support was provided by European Social Fund. If there are other authors, they declare that they have no known competing financial interests or personal relationships that could have appeared to influence the work reported in this paper.
